# Budget impact analysis of vaccination against Haemophilus influenzae type b as a part of a Pentavalent vaccine in the childhood immunization schedule of Iran

**DOI:** 10.1186/s40199-017-0166-0

**Published:** 2017-01-14

**Authors:** Fatemeh Teimouri, Abbas Kebriaeezadeh, Seyed Mohsen Zahraei, MohammadMahdi Gheiratian, Shekoufeh Nikfar

**Affiliations:** 1 Department of Pharmacoeconomics and Pharmaceutical Administration, Faculty of Pharmacy, Tehran University of Medical Sciences, Tehran, Iran; 2 Pharmaceutical Management and Economics Research Center, Tehran University of Medical Sciences, Tehran, Iran; 3 Center for Communicable Diseases Control, Ministry of Health and Education, Tehran, Iran; 4 Department of Emergency Medicine, Besat Hospital, Hamadan University of Medical Sciences, Hamadan, Iran; 5 Evidence-Based Medicine Group, Pharmaceutical Sciences Research Group, Tehran University of Medical Sciences, Tehran, Iran

**Keywords:** Budget impact analysis, Pentavalent vaccine, Haemophilus influenzae type b, Immunization schedule, Iran

## Abstract

**Background:**

Health decision makers need to know the impact of the development of a new intervention on the public health and health care costs so that they can plan for economic and financial objectives. The aim of this study was to determine the budget impact of adding Haemophilus influenzae type b (Hib) as a part of a Pentavalent vaccine (Hib-HBV-DTP) to the national childhood immunization schedule of Iran.

**Methods:**

An excel-based model was developed to determine the costs of including the Pentavalent vaccine in the national immunization program (NIP), comparing the present schedule with the previous one (including separate DTP and hepatitis B vaccines). The total annual costs included the cost of vaccination (the vaccine and syringe) and the cost of Hib treatment. The health outcome was the estimated annual cases of the diseases. The net budget impact was the difference in the total annual cost between the two schedules. Uncertainty about the vaccine effectiveness, vaccination coverage, cost of the vaccine, and cost of the diseases were handled through scenario analysis.

**Results:**

The total cost of vaccination during 5 years was $18,060,463 in the previous program and $67,774,786 in the present program. Inclusion of the Pentavalent vaccine would increase the vaccination cost about $49 million, but would save approximately $6 million in the healthcare costs due to reduction of disease cases and treatment costs. The introduction of the Pentavalent vaccine resulted in a net increase in the healthcare budget expenditure across all scenarios from $43.4 million to $50.7 million.

**Conclusions:**

The results of this study showed that the inclusion of the Pentavalent vaccine in the NIP of Iran had a significant impact on the health care budget and increased the financial burden on the government.

**Graphical abstract:**

Budget impact of including Pentavalent vaccine in the national immunization schedule of Iranᅟ
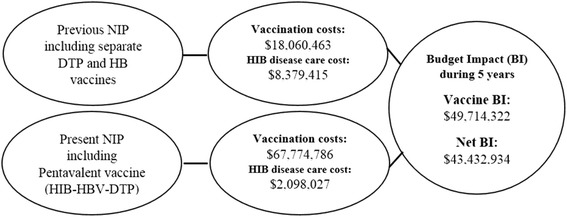

## Background

Considering the increasing costs of the health sector in all countries of the world and the limited budget in this area, it is necessary to use the resources efficiently. Due to the lack of uniformity in terms of health and financial resources in different countries, decision-makers should make decisions based on the existing conditions and the needs of their countries using evidence-based measures. Economic assessment in the context of each country is an example of evidence. Economic evaluation studies are valuable tools to demonstrate ways to use limited resources efficiently [1].

Based on the need, especially when a new technology emerges which is often expensive, economic evaluation studies, including cost-effectiveness studies and budget impact analysis (BIA) are designed and implemented and the results of these studies will be available to decision makers in the health sector. While cost-effectiveness studies demonstrate the value of an intervention and help to assign a priority to interventions, budget impact analysis helps to assess the affordability and sustainability of the interventions [1, 2]. Among these technologies are new and expensive vaccines.

Haemophilus influenzae type b (Hib) plays an important role in the mortality and morbidity of children under 5 years old all around the world. In 2000, Hib caused 8.13 million serious illnesses worldwide and 371000 deaths in children aged 1–59 months [3].

The most important clinical manifestations of Hib are meningitis, pneumonia, and other non-meningitis-non-pneumonia (NMNP) diseases like septicemia, orbital cellulitis, septic arthritis, osteomyelitis, and sinusitis [4].

The first vaccine against Hib was produced in early 1970s and the vaccines against Hib are currently available in two forms on the market, monovalent or in combination with other vaccines [5].

Some advantages of combined vaccines are reducing the number of visits and injections, reducing the patient discomfort, increasing compliance, reducing operational costs, and optimizing preventive measures. The combined vaccine including DTP-HB-HIB has shown proper immunogenicity in clinical trials with similar adverse events to the separate vaccines [6, 7].

One of the oldest and most successful public health interventions in Iran is the immunization program which has resulted in the control, elimination, or eradication of infectious diseases suggested by the World Health Organization (WHO). Iran accepted the Expanded Program of Immunization (EPI) in 1978 and implemented the EPI since 1984. Vaccination against Diphteria, Tetanus, and Pertusis has been carried out since then, and Hepatitis B vaccine was added to the national program in 1993. The national immunization technical advisory group (NITAG) was established in 1982 prior to the implementation of the EPI. The NITAG provides evidence based advice to the Ministry of Health on the national immunization policy and plays an important role in the decision making process. The evidence includes the disease burden and cost-effectiveness studies [8, 9]. To determine whether Hib vaccination should be included in the immunization program, two studies were conducted in Iran. In the first study conducted under the supervision of the WHO, it was found that the annual incidence rate of Hib meningitis was 8.5/100,000 in children under 5 years old (unpublished study).

In the second study, the specimens obtained from the oropharynx of 1000 children in Tehran were cultured from October 2005 to March 2006. The results demonstrated that the prevalence of Hib carriers was 7.6% [10]. Before adoption of this new vaccine, a cost-effectiveness study was done which concluded vaccination against Hib was cost-effective in the context of Iran [11].

Based on these studies and other factors like WHO recommendations, the Iranian Ministry of Health added the Hib vaccine as a Pentavalent vaccine in combination with DTP (Diphteria-Tetanus-Pertusis) and Hepatitis B to the immunization program from November 2014 instead of separate Hepatitis B and DTP vaccines. This vaccine is available to all children who are born in Iran and is administered in 3 doses at 2, 4, and 6 months of age.

Health decision makers need to know the impact of new vaccines on the public health and health care costs so that they can plan for economic and financial objectives. Financial sustainability for immunization programs requires resources that are available at the right time to achieve the desired health goals ultimately. Lack of or delay in the planned budget, limits the population covered in the immunization program; therefore, a sufficient, timely, and reliable budget supply is very vital.

BIA studies predict funds needed for vaccine supply, help with proper budget planning, and give clear visibility to decision makers and health planners.

The aim of this study was to determine the budget impact of adding the Pentavalent vaccine (HIB-HBV-DTP) to the national childhood immunization schedule in Iran. In this regard, we estimated the annual health benefits of the vaccine and then estimated the impact of introducing the Pentavalent vaccine to the national childhood immunization program on the health budget.

## Methods

### Study design

Since we previously had DTP and Hepatitis B vaccines in the national immunization schedule, we only considered the diseases caused by Hib in this study.

The health benefit of the vaccination was the difference in the annual disease cases between the two immunization schedules (with and without the Hib vaccine). To assess the impact of the Pentavalent vaccine on the health budget, we estimated the annual direct medical costs of the treatment of Hib diseases in the previous immunization schedule (without the Pentavalent) and the present schedule (including Pentavalent), so the comparison arm was the previous schedule. The perspective of the study was the government and governmental and quasi-governmental insurance organizations. Total annual costs were the treatment costs of Hib diseases and vaccination costs. Discounting was not applied to the budget impact analysis because the budget holder is interested in real financial streams over time and it is intended as a year-by-year projection to guide budgetary planning [1]. We used the Microsoft Excel^®^ 2013 for all the calculations.

The time horizon was 5 years from November 2014 to November 2019. The study population was all the children born in this period and the first birth cohort was the children that had their first vaccine dose in November 2014. The health outcomes were the estimated annual cases of meningitis, pneumonia, and septicemia as NMNP diseases caused by Hib and also the complications of meningitis, including seizure disorders, hearing problems, deafness, and cerebral palsy.

According to the official exchange price in 2015–2016, the mean price of the $1 was 29721 Iranian Rials [12].

### Vaccination costs

To estimate the vaccination costs, we considered the costs of the vaccine and the syringe. Since the Pentavalent vaccine replaced the DTP vaccine in the schedule (at 2, 4 and 6 months of age), we did not include the logistic and personnel costs because they were the same in both programs. We did not consider the adverse effects of the vaccine because in two systematic reviews on the efficacy and safety of Hib conjugate vaccines including both monovalent and combined vaccines, no serious adverse effects were reported in any of the included trials [13, 14]. Moreover, after consultation with experts in the Iranian Ministry of Health, we assumed the adverse effects would be similar to the DTP vaccine (injection site reactions and a mild fever) [15].

$$ \begin{array}{l}\mathrm{Total}\ \mathrm{annual}\ \mathrm{vaccine}\ \mathrm{costs} = \mathrm{Cost}\ \mathrm{per}\ \mathrm{vaccine}\ \mathrm{dose} \times \mathrm{predicted}\ \mathrm{vaccination}\ \mathrm{coverage} \times \\ {}\mathrm{target}\ \mathrm{population} \times \mathrm{number}\ \mathrm{of}\ \mathrm{dose}\mathrm{s}/\mathrm{child} \times \mathrm{predicted}\ \mathrm{wastage}\ \mathrm{factor}\end{array} $$

Table 1 shows the information of the vaccines in the two schedules. In addition, the cost of each syringe was assumed $0.047 and the number of needed syringes per child was considered 5 in the previous and 3 in the present schedule. The information about the costs of the vaccine and syringe and the estimated wastage rate of each vaccine (Pentavalent, DTP and Hepatitis B) was obtained from the Iranian Ministry of Health. The vaccination coverage was obtained from a national survey and considered similar to the DTP vaccine coverage in Iran with different rates in urban and rural districts [16].

**Table 1:** Vaccines information used to calculate cost of vaccination in 2015

Vaccine	Average wastage (%)	Cost per dose (USD)	Cost per dose including wastage (USD)	Number of doses per FIC	Cost per FIC including wastage (USD)	Vaccination coverage (%)
Pentavalent (single dose)	2	2.85	2.91	3	8.73	96.47
Pentavalent (multi dose)	10	2.34	2.60	3	7.8	96.54
HB	15	0.33	0.39	3	1.17	96.20
DTP	20	0.31	0.38	3	1.14	96.51

*DTP* Diphteria, Tetanus, Pertusis, *HB* Hepatitis B, *FIC* Fully Immunized Child, *USD* United States Dollars

We obtained the target population data from the Statistical center of Iran [17] and estimated the birth cohort based on the population growth rate announced by the same center for the next four years.

The policy of the Ministry of Health to reduce the vaccine wastage rate is to use single dose Pentavalent vaccines in the rural districts and multi dose Pentavalent vaccines in the urban districts of the country. We considered the costs of these two kinds of vaccines and the different populations in urban and rural districts of Iran in our calculations.

### Cost estimates (direct medical costs)

To obtain the direct medical costs of the treatment of meningitis, pneumonia, septicemia, seizure disorders, and cerebral palsy due to meningitis, we used the mean costs of hospitalization for children less than 5 years of age from two public hospitals in Tehran (Markaz Tebi and Rasoul Akram Hospitals) from March 2015 to March 2016. To estimate the medical costs of hearing loss and deafness (including cochlear implantation), we used the experts’ opinions (Audiometrists & otolaryngologists).

For patients who had seizure during their hospitalization with a permanent sequelae on brain images, we added a cost of two years of outpatient seizure therapy based on the expert opinion.

The Ministry of Health (government) is responsible for providing free vaccines for all the children in the country and there are several governmental and quasi-governmental (Social Security Insurance, Iran Health Insurance, and Armed Forces Medical Services Insurance) insurance organizations in Iran [18]. Ninety-seven percent of the Iranian population has health insurance and 90% has governmental and quasi-governmental insurance, and the health insurance contribution for hospitalized patients is 90% [19], so the perspective of this study was the government and governmental and quasi-governmental insurance organizations [20, 21]. The tariffs for drugs and medical services were considered the same in these insurance organizations.

### Disease burden

Based on a study conducted under the supervision of the WHO, which has not been published yet, the annual incidence rate of Hib meningitis, before the vaccination era was 8.5/100,000 in children under 5 years in Iran.

Based on the CCDC information, we assumed 80% of meningitis cases were children under 1 year of age and estimated Hib meningitis cases in the five-year period of the study.

For pneumonia cases, according to the results of rapid Hib disease assessment method proposed by the WHO, there are 5 cases of Hib pneumonia for each case of Hib meningitis [3].

Since other Hib diseases are relatively rare, these are grouped in one syndrome as NMNP Hib diseases and the rates between cases of NMNP and meningitis assumed 0.35 [4]. For assessing the consequences of Hib meningitis, we considered complications seen within the first year. The probability of hearing loss, deafness, seizure disorders, and cerebral palsy was considered 12.5%, 3.2%, 5%, and 3.5% respectively according to 2 existing meta- analyses and a domestic study [22–24]. The efficacy of the Hib vaccine after 3 doses against confirmed invasive Hib diseases, meningitis, and pneumonia was considered 93%, 88%, and 67%, respectively [25].

### Sensitivity analysis

In order to minimize model uncertainty, a sensitivity analysis was performed with alternative scenarios. In the basic scenario, we considered that the cost of the vaccines and treatment of Hib diseases would be constant during 5 years and the vaccine effectiveness would be 93%, 88%, and 67% against confirmed invasive Hib diseases, meningitis, and pneumonia, respectively based on a meta-analysis [25].

In the second scenario, we assumed that the cost of the Pentavalent vaccine would increase (based on information obtained from Iran Food and Drug Administration (IFDA)) during 5 years, the treatment costs would increase by 12% annually (the median increase in the cost of hospital records in the second year). The vaccine effectiveness was assumed the same as the basic scenario.

In the third scenario, we assumed that the cost of vaccination and the cost of treatment would increase during 5 years and the vaccine effectiveness would be 93% against all Hib diseases. In the fourth scenario, we assumed that the costs would increase during 5 years, the vaccine effectiveness was considered the same as the basic scenario, but the vaccine coverage was assumed to be 100%.

## Results

Table 1 shows what information was used to estimate the costs of vaccination. In the previous schedule, 3 doses of the DTP vaccine were administered at 2, 4, and 6 months of age and the hepatitis B vaccine was administered at birth and at 2 and 6 months of age. Therefore, the Pentavalent vaccine replaced 3 doses of the DTP vaccine and 2 doses of the hepatitis B vaccine but we still have the hepatitis B vaccine at birth. Based on the information from the Statistical center of Iran, to estimate the rural and urban population of Iran, we assumed that 77% of the annual cohort birth was in urban districts and 23% was in rural districts of the country. Our first cohort was 1,534,362 children of whom 1,181,458 were in urban and 352,903 were in rural districts. The vaccination coverage for the Pentavalent single dose, Pentavalent multi dose, hepatitis B, and DTP was 96.47%, 96.54%, 96.2%, and 96.51%, respectively [16]. The cost of fully immunizing a child including the wastage rate was $8.73 for the Pentavalent single dose, $7.8 for the Pentavalent multi dose, $1.17 for the hepatitis B, and $1.14 for the DTP vaccine.

The vaccination cost of previous immunization schedule and the present schedule are shown in Table 2. The total cost of vaccination during 5 years was $18,060,463 in the previous program and $67,774,786 in the present program, so inclusion of the Pentavalent vaccine increased the vaccination cost by about $49 million.

**Table 2:** Vaccination costs including vaccine and syringe costs for all children under 5

	HB & DTP vaccine costs (USD)	HB & DTP vaccine & syringes costs (USD)	Pentavalent vaccine costs (USD)	Pentavalent vaccine & syringes costs (USD)
Year 1	2,873,626	3,221,945	11,881,606	12,090,866
Year 2	3,017,308	3,383,042	12,475,686	12,695,409
Year 3	3,189,294	3,575,875	13,186,800	13,419,047
Year 4	3,393,409	3,804,731	14,030,756	14,277,866
Year 5	3,634,341	4,074,867	15,026,939	15,291,595
Total	16,107,980	18,060,463	66,601,789	67,774,786

*DTP* Diphteria, Tetanus, Pertusis, *HB* Hepatitis B, *USD* United States Dollars

Table 3 shows the estimated treatment costs of Hib diseases in children less than 5 years of age obtained from hospital records and expert opinions.

**Table 3:** Estimated Hib disease treatment costs in children less than 5 years

Hib diseases	Mean cost (USD)	SD (USD)	95% CI
Meningitis	693	393	(401–984)
Pneumonia	387	162	(257–517)
Septicemia	719	488	(380–1057)
Seizure disorder	570	98	(433–707)
2 years outpatient seizure disorder^a^	418	NA	NA
Hearing loss^a^	42	NA	NA
Deafness^a^	1773	NA	NA
Cerebral palsy	554	230	(294–814)

*Hib* Haemophilus influenzae type b, *NA* Not Applicable, *USD* United States Dollars; ^a^: Based on expert opinions

Table 4 shows the estimated Hib disease cases before and after the inclusion of the Pentavalent vaccine in the childhood immunization schedule in 5 years. The Pentavalent vaccine would approximately prevent 3010 cases of meningitis, 11458 cases of pneumonia, and 114 cases of septicemia during 5 years.

**Table 4:** Estimated Hib disease cases before and after inclusion of Pentavalent vaccine in the childhood immunization schedule in 5 years

Hib diseases	No Pentavalent vaccine	With Pentavalent vaccine	Estimated event averted
Meningitis	3420	410	3010
Pneumonia	17100	5642	11458
Septicemia	1197	83	1114
Seizure disorder	120	14	106
Hearing loss	427	53	374
Deafness	109	13	96
Cerebral palsy	171	21	150

*Hib* Haemophilus influenzae type b

Based on the estimated number of patients and the cost of treatment per patient, the overall cost of treating patients was calculated. Before the inclusion of the Pentavalent vaccine, the total treatment cost of Hib diseases during 5 years was $8,379,415 which reduced to $2,098,027 afterwards. Therefore, the financial saving would be about $6 million.

As Table 5 shows, the cost of new vaccination program was much higher for the government. In the first year, in the previous schedule, the cost of vaccination was estimated to be about $3 million but the new vaccination program cost about $12 million (approximately 4 times more). The same also applied in following years. On the other hand, the inclusion of this vaccine saved approximately $6 million in healthcare costs over 5 years. During 5 years of immunization, the incremental cost was $43,432,934, from $7,897,693 in the first year to $9,714,167 in the fifth year.

**Table 5:** Budget impact results

Annual cost outcomes	Year 1	Year 2	Year 3	Year 4	Year 5	Total (5 years results)
Previous vaccination program (DTP + HB)						
Vaccination costs (USD)	3,221,945	3,383,042	3,575,875	3,804,731	4,074,867	18,060,463
Hib disease care costs (USD)	1,295,624	1,522,358	1,697073	1,859,932	2,004,425	8,379,415
Total costs (USD)	4,517,569	4,905,401	5,272,949	5,664,664	6,079,293	26,439,878
New vaccination program (Pentavalent)						
Vaccination costs (USD)	12,090,866	12,695,409	13,419,047	14,277,861	15,291,595	67,774,786
Hib disease care costs (USD)	324,396	381,166	424,911	465,687	501,865	2,098,027
Total costs (USD)	12,415,263	13,076,575	13,843,959	14,743,554	15,793,461	69,872,813
Budget impact (BI)						
Vaccine BI (USD)	8,868,921	9,312,367	9,843,172	10,473,135	11,216,727	49,714,322
Hib disease care BI (USD)	-971,227	-1,141,192	-1,272,162	-1,394,245	-1,502,559	-6,281,387
Net BI (USD)	7,897,693	8,171,174	8,571,009	9,078,889	9,714,167	43,432,934

*DTP* Diphteria, Tetanus, Pertusis, *HB* Hepatitis B, *USD* United States Dollars, *Hib* Haemophilus influenzae type b

### Sensitivity analysis

As shown in Table 6, all scenarios showed that the inclusion of the Pentavalent vaccine in the national immunization schedule of Iran increased the financial burden on the government. During 5 years, the total net budget impact results ranged from + $43 million in the basic scenario to + $50 million in the fourth scenario, assuming a vaccination coverage rate of 100% in both comparative arms. The total net budget impact was + $48 million in the second scenario where the cost of vaccination and disease care increased during 5 years, which is closer to reality, and + $46 millions in the third scenario with more effective vaccines.

**Table 6:** Sensitivity analysis results

	Year 1	Year 2	Year 3	Year 4	Year 5	Total (5 years results)
Base scenario						
Vaccine BI (USD)	8,868,921	9,312,367	9,843,172	10,473,135	11,216,727	49,714,322
Hib disease care BI (USD)	-971,227	-1,141,192	-1,272,162	-1,394,245	-1,502,559	-6,281,387
Net BI (USD)	7,897,693	8,171,174	8,571,009	9,078,889	9,714,167	43,432,934
Second scenario						
Vaccine BI (USD)	8,868,921	9,940,023	11,269,731	12,577,727	14,222,115	56,878,518
Hib disease care BI (USD)	-971,227	-1,278,135	-1,595,800	-1,958,814	-2,364,306	-8,168,285
Net BI (USD)	7,897,693	8,661,887	9,673,930	10,618,913	11,857,808	48,710,233
Third scenario						
Vaccine BI (USD)	8,868,921	9,940,023	11,269,731	12,577,727	14,222,115	56,878,518
Hib disease care BI (USD)	-1,204,777	-1,585,487	-1,979,541	-2,429,848	-2,932,849	-10,132,504
Net BI (USD)	7,664,143	8,354,535	9,290,189	10,147,879	11,289,266	46,746,014
Fourth scenario						
Vaccine BI (USD)	9,183,753	10,293,241	11,670,517	13,025,331	14,728,569	58,901,414
Hib disease care BI (USD)	-971,227	-1,278,135	-1,595,800	-1,958,814	-2,364,306	-8,168,285
Net BI (USD)	8,212,525	9,015,105	10,074,716	11,066,517	12,364,262	50,733,128

*BI* Budget Impact, *USD* United States Dollars

## Discussion

The present study was the first BIA for vaccines in Iran with the aim of evaluating the financial consequences of adding the Pentavalent vaccine (DTP-HB-HIB) to the national immunization schedule. Separate DTP and hepatitis B vaccines were replaced by the Pentavalent in the national immunization schedule. We measured the treatment costs of Hib diseases (meningitis, pneumonia, and septicemia) and also the costs of acute meningitis sequelae (seizure disorder, hearing problems, deafness, and cerebral palsy). The study found that the average per case treatment cost of meningitis, pneumonia, and septicemia, the main manifestations of Hib, was $693, $387, and $719 respectively. Moreover, considering the acute and permanent sequelae, meningitis was the costliest Hib disease. Using hospital records as well as the experts in calculating costs, can be seen as an advantage in our study.

The total cost of vaccination during 5 years was $18,060,463 in the previous program and $67,774,786 in the present program, so inclusion of the Pentavalent vaccine increased the vaccination cost by about $49 million but saved approximately $6 million in the healthcare costs due to reduction of disease cases and treatment costs.

Scenario analyses were developed considering uncertainties regarding vaccine effectiveness, cost of the diseases and vaccine, and vaccination coverage. The net budget impact during 5 years was + $43 million in the basic scenario, +$48 million in the second scenario in which the treatment costs and vaccine cost were assumed to increase during 5 years, +$46 million in the third scenario in which the costs were assumed to increase during 5 years and the vaccine was assumed to be more effective, and + $50 million in the last scenario with a 100% vaccination coverage. As the results showed, the difference between the best and the worst scenario was 16%. The sensitivity analysis showed that the model results were robust to changes in the parameters analyzed and the uncertainties in this study did not have significant effects on the results. The four scenarios analyzed in our model suggest that after 5 years, adding the Pentavalent vaccine to the national immunization schedule would impose a significant burden on the governmental healthcare budget.

The most important reason for this financial burden is the price of the vaccine. Before including the Pentavalent vaccine, the program offered universal immunization against tuberculosis, poliomyelitis, diphtheria, tetanus, pertussis, hepatitis B, measles, mumps, and rubella [9]. The replacement of the domestic vaccines (DTP and hepatitis B) with expensive imported vaccines (Pentavalent) increased the net budget impact, so regarding the history and potential for vaccine production in the country, one solution to reduce this financial burden in the long term is to support domestic production by investment in this field, renewal of the infrastructures, and implementation of new technologies.

In the Iranian healthcare system, the Ministry of Health is responsible for providing free vaccines for all children in the country. Every year, the Management and Planning Organization of Iran approves the budget needed for vaccine supplies. The purchase and procurement of vaccines for national programs is the responsibility of the IFDA. The IFDA estimates the maximum amount of funding every year. According to the existing documents at IFDA, in the second year of including the Pentavalent vaccine in the program, by assuming a 100% vaccination coverage rate, the IFDA estimated the maximum budget needed for purchasing Pentavalent vaccine would be $14 million which is similar to our findings ($13,800,000). However, BIA has never been conducted by the Iranian MOH. The results of this study can be used for budget planning, especially by the Management and Planning Organization of Iran.

Based on domestic studies on the epidemiology of Hib meningitis and the cost-effectiveness of the Hib vaccine in Iran (previously described), WHO recommendations, availability of the vaccine in other countries, and the efficacy of the vaccine, the NITAG approved Hib vaccination to be included in the EPI. However, some issues like financing problems, different priorities of the Ministry of Health, plans to produce the Hib vaccine in the country, and finding a proper importer caused a delay in the inclusion of the vaccine in the immunization schedule [9]. Based on WHO reports, Iran was one of the last countries to include the Hib vaccine in its national vaccination program.

In other countries, the Hib vaccine was introduced sooner and many cost-effectiveness studies were done about Hib vaccination which all concluded that this intervention was cost-effective in the context of their countries like India, Indonesia, Kenya, and Russia [26–29]. However, for very new vaccines like rotavirus vaccine and pneumococcal vaccine, BIA studies have been done in different countries like Spain [30, 31], Germany [32], and New Zealand [33]. The BIA is a new decision making tool and since the Hib vaccine is almost an old vaccine, no BIA study has evaluated it. Two BIA studies have been conducted for including the rotavirus vaccine in the childhood immunization schedule in Spain and New Zealand. The Spanish study estimated that the economic impact of introducing universal infant rotavirus vaccination in Spain would be €10.43 million per year, from the perspective of the Spanish National Health System [30]. In New Zealand, the budget impact in the 5^th^ year of an immunization program with a Pentavalent rotavirus vaccine was estimated $4.78 million from the perspective of the government [33].

As this study was the first BIA on vaccines in Iran, its methodology may be useful for future BIA studies on other new vaccines because all the pharmaceutical companies have to submit an economic evaluation profile (including the cost-effectiveness study and BIA) for their new products to the IFDA from 2014, and the vaccines are not an exception [34].

Our study has some limitations. To conduct accurate, useful, and attributable economic evaluation studies like BIA, reliable information about the disease burden is required. Lack of disease burden studies and reliable information on the incidence of Hib diseases and disease cases were the main limitations of this study. One of the reasons for the lack of the above information is that when a patient is hospitalized with meningitis symptoms, antibiotic therapy starts immediately so the culture of the specimens taken from the patient will be negative. On the other hand, because the PCR method is not available in all hospitals, it is difficult to identify the pathogen.

The second limitation was the lack of vaccine efficacy data in Iran. We extracted the vaccine efficacy data from a meta-analysis [25]. However, we can not predict whether the imported Pentavalent vaccine will have the same efficacy in Iran, and more data need to be collected on the effectiveness and safety of the Pentavalent vaccine in Iran.

Another point is that in measuring the treatment costs of complications due to meningitis, we considered the problems which can be seen in the first year (acute complications). However, permanent sequelae, like mental retardation, cause productivity loss and health costs in the long term, so the inclusion of the Hib vaccine will save some costs from the perspective of the society.

To respond to domestic demands and keep pace with other countries, inclusion of new and expensive vaccines in the NIP of Iran is inevitable. Despite the limitations mentioned above, the results suggest an increase in the government expenditure on new vaccines. However, it should be noted that the BIA is one of the decision-making tools and other reasons may affect the policy makers’ decisions on the arrival of the new vaccines. One of the most important reasons can be the value of the children’s lives as the productive forces in the future. It is suggested to conduct a study to investigate the reasons affecting the decision-makers’ decision on the arrival of new vaccines.

## Conclusion

In conclusion, according to our model and estimates, the results showed that inclusion of the Pentavalent vaccine (HIB-HBV-DTP) in the national immunization schedule of Iran causes a significant impact on the healthcare budget and increases the financial burden on the government.

## Abbreviations

BIA: Budget Impact Analysis
CCDC: Center for Communicable Disease Control
DTP: Diphteria-Tetanus-Pertusis
EPI: Expanded Program of Immunization
HB: Hepatitis B
HIB: Haemophilus influenzae type b
IFDA: Iran Food and Drug Administration
NDL: National Drug List
NIP: National Immunization Program
NITAG: National Immunization Technical Advisory Group
NMNP: Non-Meningitis-Non-Pneumonia
WHO: World Health Organization
